# Comparison of LASSO and random forest models for predicting the risk of premature coronary artery disease

**DOI:** 10.1186/s12911-023-02407-w

**Published:** 2023-12-20

**Authors:** Jiayu Wang, Yikang Xu, Lei Liu, Wei Wu, Chunjian Shen, Henan Huang, Ziyi Zhen, Jixian Meng, Chunjing Li, Zhixin Qu, Qinglei he, Yu Tian

**Affiliations:** 1 School of Nursing, Liaoning University of Traditional Chinese Medicine, 110847 Shenyang, China; 2 Department of Cardiovascular Medicine, The Second Affiliated Hospital of Shenyang Medical College, 110002 Shenyang, China; 3 Institute of Humanities and Social Sciences, Shenyang University, 110044 Shenyang, China; 4 Department of Cardiac Surgery, The Second Affiliated Hospital of Shenyang Medical College, 110002 Shenyang, China; 5 Library, Shenyang Medical College, 110034 Shenyang, China; 6 School of Public Health, Shenyang medical college, 110034 Shenyang, China; 7 School of nursing, Liaoning Jinqiu Hospital, 110034 Shenyang, China; 8 School of nursing, The First Affiliated Hospital of China Medical University, 110034 Shenyang, China; 9 School of nursing, Shenyang medical college, 110034 Shenyang, China

**Keywords:** Premature coronary artery disease, Lasso, Random forest, Risk prediction

## Abstract

**Purpose:**

With the change of lifestyle, the occurrence of coronary artery disease presents a younger trend, increasing the medical and economic burden on the family and society. To reduce the burden caused by this disease, this study applied LASSO Logistic Regression and Random Forest to establish a risk prediction model for premature coronary artery disease(PCAD) separately and compared the predictive performance of the two models.

**Methods:**

The data are obtained from 1004 patients with coronary artery disease admitted to a third-class hospital in Liaoning Province from September 2019 to December 2021. The data from 797 patients were ultimately evaluated. The dataset of 797 patients was randomly divided into the training set (569 persons) and the validation set (228 persons) scale by 7:3. The risk prediction model was established and compared by LASSO Logistic and Random Forest.

**Result:**

The two models in this study showed that hyperuricemia, chronic renal disease, carotid artery atherosclerosis were important predictors of premature coronary artery disease. A result of the AUC between the two models showed statistical difference (*Z* = 3.47, *P* < 0.05).

**Conclusions:**

Random Forest has better prediction performance for PCAD and is suitable for clinical practice. It can provide an objective reference for the early screening and diagnosis of premature coronary artery disease, guide clinical decision-making and promote disease prevention.

**Supplementary Information:**

The online version contains supplementary material available at 10.1186/s12911-023-02407-w.

## Introduction

Coronary artery disease (CAD) has become a leading cause of mortality in many countries. Although the mortality rate of CAD has declined in developed countries, it is rising in developing countries or countries in economic transition [1]. According to the Global Burden of Disease report, about 9.14 million people worldwide died from CAD in 2019 [2]. The increase in deaths in China accounts for about 38.2% of the global increase in deaths from CAD [3, 4]. As the population of CAD patients becomes younger, the third National Cholesterol Education Program Adult Treatment Group Guidelines (NECP ATP III) defines men < 55 years and women < 65 years with CAD as having premature coronary artery disease (PCAD) [5]. The US NHIS data indicate that among Asian Indians and “other Asians” prevalence of PCAD higher than Whites adults [6, 7]. Because there are few typical symptoms before the onset of PCAD, it is often not diagnosed or misdiagnosed [8]. Studies have confirmed that the degree of fibrosis of early-onset coronary plaques is higher than that of late-stage coronary artery plaques [9]. As a key event in the inflammatory process of atherosclerosis, fibrosis participates in the regulation of plaque stability, and the instability of plaques causes thrombosis. If it is not treated in time, it is very likely to be life threatening [10]. Therefore, early screening for PCAD is the key to guiding clinical decision-making and promoting disease prevention.

The Framingham risk assessment model is a classic cardiovascular disease assessment tool widely recognized domestically and in foreign countries. However, some studies have pointed out that this model cannot effectively predict the incidence risk of PCAD in healthy young people with family history [11]. Although coronary angiography is the gold standard for the diagnosis of PCAD, it is not suitable as an early detection tool for asymptomatic people because of its high price, invasive nature, and potential for allergic reactions to the contrast agent. At present, researchers in many countries have attempted to identify predictors of cardiovascular disease, and some reports suggest that factors such as C-reactive protein, hypercholesterolemia, high-density lipoprotein cholesterol, family history of CAD, and smoking can predict PCAD risk in patients [12–15]. For these predictors, a variety of cardiovascular risk assessment tools have been developed and improved. However, a PCAD risk prediction model has yet to be developed. Therefore, the establishment of an accurate prediction model of PCAD that can reduce unnecessary invasive examinations of patients and ensure effective screening and diagnostic ability for PCAD has become a research hotspot.

In this study, we constructed a risk prediction model for PCAD that is based on traditional logistic regression analysis. However, the conventional linear regression cannot solve the problem of data collinearity. Therefore, we used a machine learning method called LASSO to reduce the dimension and deal with variable collinearity. Machine learning can better mine higher dimensions, complex structures, and essential medical data compared with traditional statistical methods [16, 17]. Random forest (RF) models can handle the problem of nonlinearity and data loss and assign importance scores to each feature variable in classification to screen for the variable that plays an essential role in the category [18, 19]. At the same time, the RF approach does not need to consider multivariate collinearity or make a variable selection. The purpose of this study was to compare the LASSO logistic regression and RF methods for predicting the risk PCAD and to develop a practical and applicable risk prediction model.

## Methods

We conducted this retrospective study to construct and validate a risk prediction model for PCAD and used STROBE and TRIPOD as a guide [20, 21]. This study was approved by the ethics committee of The Second Affiliated Hospital of Shenyang Medical College (2022-Shen Medical second hospital-019). Along with confirmation that the study complies with all regulations and confirmation that informed consent was obtained.

### Data sources

The data are from September 2019 to December 2021. We screened electronic data for cases in the Department of Cardiology ward of a class III hospital in Liaoning Province, China. Finally, 1004 patients were confirmed.

### Study population

**Fig. 1:**
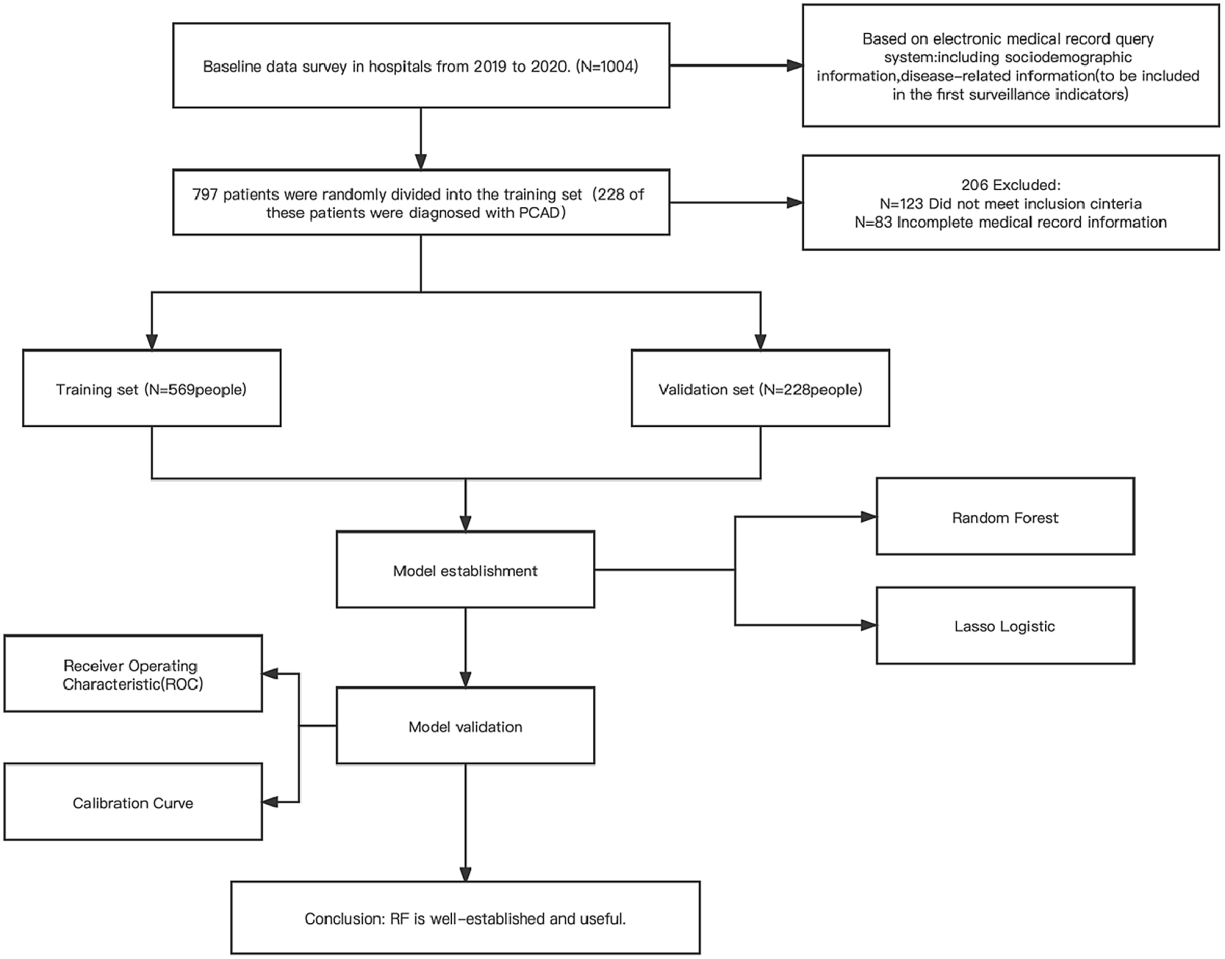
Study cohort and exclusions

Inclusion criteria: All patients diagnosed with CAD who visited the cardiology ward from September 2019 to December 2021. Exclusion criteria:patients with severe cognitive impairment, comorbidity with other serious diseases, previous coronary artery bypass graft treatment or heart transplantation, chest pain such as suspected aortic coarctation, and pulmonary embolism were excluded. The final loss was 20.5%. The data from 797 patients were ultimately evaluated. 226 of these patients were diagnosed with PCAD. (The process of the study design was shown in Fig. 1).

### Candidate variables

We extracted sociodemographic, disease-related, and laboratory-related data from the patients’ medical records. Continuous variables included age, systolic blood pressure, diastolic blood pressure,Kalium(K), chlorine(Cl),urea, creatinine, total cholesterol (TC), fasting plasma glucose, low-density lipoprotein cholesterol (LDL-C), high-density lipoprotein cholesterol (HDL-C), and triglycerides (TG) levels. Categorical variables included sex, smoking history, and alcohol consumption history, surgical history, diabetes, hypertension, overweight, chronic kidney disease(CKD), carotid artery atherosclerosis (CAA), hyperuricemia (HUA), Hyperlipemia.

Definition and measurement of relevant indicators were as follows. Overweight was defined as body mass index ≧ 24.0 kg/m^2^. HUA was defined as nondaily fasting blood UA levels > 420 mol/L in males and 360 mol/L in females under normal purine diet status [22]. Chronic kidney disease was defined as structural or functional kidney abnormalities with health effects for > 3 months [23]. The 2007 Chinese guidelines for the prevention and treatment of dyslipidemia for hyperlipidemia were as follows: TC > 5.18 mmol/L, LDL-C 3.37 mmol/L, HDL-C < 1.04 mmol/L, TG ≥ 1.7 mmol/L [24]. Hcy≥15 mol/L serves as the diagnostic criterion for hyperhomocysteinemia (Hhcy). The criteria for CAA are based on carotid intra-media thickness: < 1.0 mm means that the patient has no carotid stiffness, l.0–1.5 mm indicates an irregular bulge of the thickened wall of the inner middle membrane, and 1.5 mm means atherosclerosis and simultaneous alteration of various arterial structures such as lumen protrusion [25].

### Grouping standard

According to the World Health Organization/International Society of Cardiology CAD is defined as: i) > = 50% stenosis, ii) involvement of at least one main coronary artery, in particular, left main trunk, anterior descending branch [6]. And NECP ATP III PCAD is defined that males < 55 or female < 65 [5]. In conclusion,all 3 criteria must be met to define PCAD.

### Data analysis

Statistical analysis was performed using R software (version 4.0.3). Measurement data consistent with the normal distribution are represented as mean ± standard deviation and were compared using two independent sample t-tests. We used the rank-sum test when data did not conform to the normal distribution (*P*_25_, *P*_75_) description. The count data is expressed in frequency percentage (%). The “caret” package was applied to group 797 participants 7:3 randomly, including 569 in the training set and 228 in the validation set.

### Model construction, comparison, and validation

All continuous variables were standardized before LASSO.We constructed the LASSO model and then screened the predictors using the LASSO regression in the “glmnet” package. LASSO can screen the variables and reduce the complexity of the model through a series of parameters, thereby avoiding overfitting. The complexity of LASSO is controlled by λ, which ultimately produces a model with fewer variables. The k-fold cross-validation was run using R software (10-fold cross-validation in this study), and lambda (λ) values were calculated, with the value with the smallest error serving as the criterion for screening predictors. Variables selected by LASSO were then subjected to logistic regression using the “rms” package. Use the “regplot” package to build the nomogram model.

The RF model was built using the “Random Forest” package. The RF model includes all predictive variables, draws samples from the database using the bootstrap resampling method, and uses the decision tree to model each set of bootstrap samples and combine multiple decision trees. Two parameters (ntree and mtry) play important roles in establishment of the model. The RF model needs to be debugged to optimize its effect to reduce the prediction error rate. We used the “caret” software package to rank the observed importance of the variables in the model following the rule that the greater the decrease in accuracy, the more significant the role of the variable in the prediction accuracy.

Finally, both models were evaluated for prediction performance using the validation set. The “pROC” package was used to generate receiver operating characteristic (ROC) curves, and the “rms” package was used to generate calibration curves.The ROC differences between two models was analyzed by the DeLong method [26].

## Results

In our study, 797 individuals met the inclusion criteria; The 135 male PCAD patients (45–55 years old) and the 93 female PCAD patients (43–65 years old). The 389 male non-PCAD patients (36–88 years old) and the 180 female non-PCAD patients (32-88years old). They were randomly divided into a training set and a validation set in a 7:3 ratio, with 569 patients in the training set and 228 patients in the validation set. All patients had completed the relevant examination. The primary characteristics of the patients in the two sets are listed in Table 1.

**Table 1:** Baseline characteristics of the study cohort

Characteristics	classify	PCAD(n = 228)	non-PCAD(n = 569)	*P*-value
Age		64.78 ± 11.24	56.64 ± 6.31	< 0.001
Sex	Male	135(59.1%)	389(68.2%)	0.01
	Female	93(41.3%)	180(32.2%)	
Overweight	yes	80(35.2%)	165(29.2%)	0.11
TG		1.75 ± 1.72	1.84 ± 1.28	0.10
HDL-C		1.68 ± 6.55	1.21 ± 0.41	0.29
LDL-C		3.14 ± 1.05	3.80 ± 14.49	0.28
TC		4.87 ± 1.35	4.87 ± 1.35	0.67
K		5.80 ± 24.46	12.49 ± 169.95	0.36
Cl		102.16 ± 7.64	101.74 ± 10.39	0.52
Urea		6.10 ± 2.21	6.13 ± 12.75	0.95
Cr		78.43 ± 33.25	78.01 ± 31.56	0.87
FPG		7.40 ± 3.27	7.07 ± 2.87	0.17
SBP		148.31 ± 26.81	150.58 ± 21.36	0.21
DBP		85.29 ± 16.37	86.55 ± 15.11	0.30
Surgical history	yes	63(28.2%)	121(21.3%)	0.05
Smoking	yes	64(28.3%)	194(34.4%)	0.10
Drinking	yes	55(24.5%)	154(27.3%)	0.38
Hypertension	yes	176(77.4%)	367(64.4%)	< 0.001
DM	yes	98(43.3%)	203(36.3%)	0.12
HUA	yes	64(28.6%)	36(6.2%)	< 0.001
CKD	yes	66(29.5%)	26(4.1%)	< 0.001
Hhcy	yes	82(36.6%)	108(19.2%)	< 0.001
CAA	yes	154(68.2%)	100(18.3%)	< 0.001
Hyperlipemia	yes	47(21.1%)	113(20.2%)	0.81

TG = triglycerides; HDL-C = high-density lipoprotein-cholesterol; LDL-C = low-density lipoprotein-cholesterol; TC = total cholesterol; Cr = creatinine; FPG = fasting plasma glucose; SBP = systolic blood pressure; DBP = diastolic blood pressure; DM = diabetes; AF = atrial fibrillation; HUA = hyperuricemia; CK-MB = creatine kinase isoenzyme; Hhcy = Hyperhomocysteinemia; CAA = carotid artery atherosclerosis; CKD = Chronic renal diseas; Cl = chlorine; K = Kalium

**Fig. 2:**
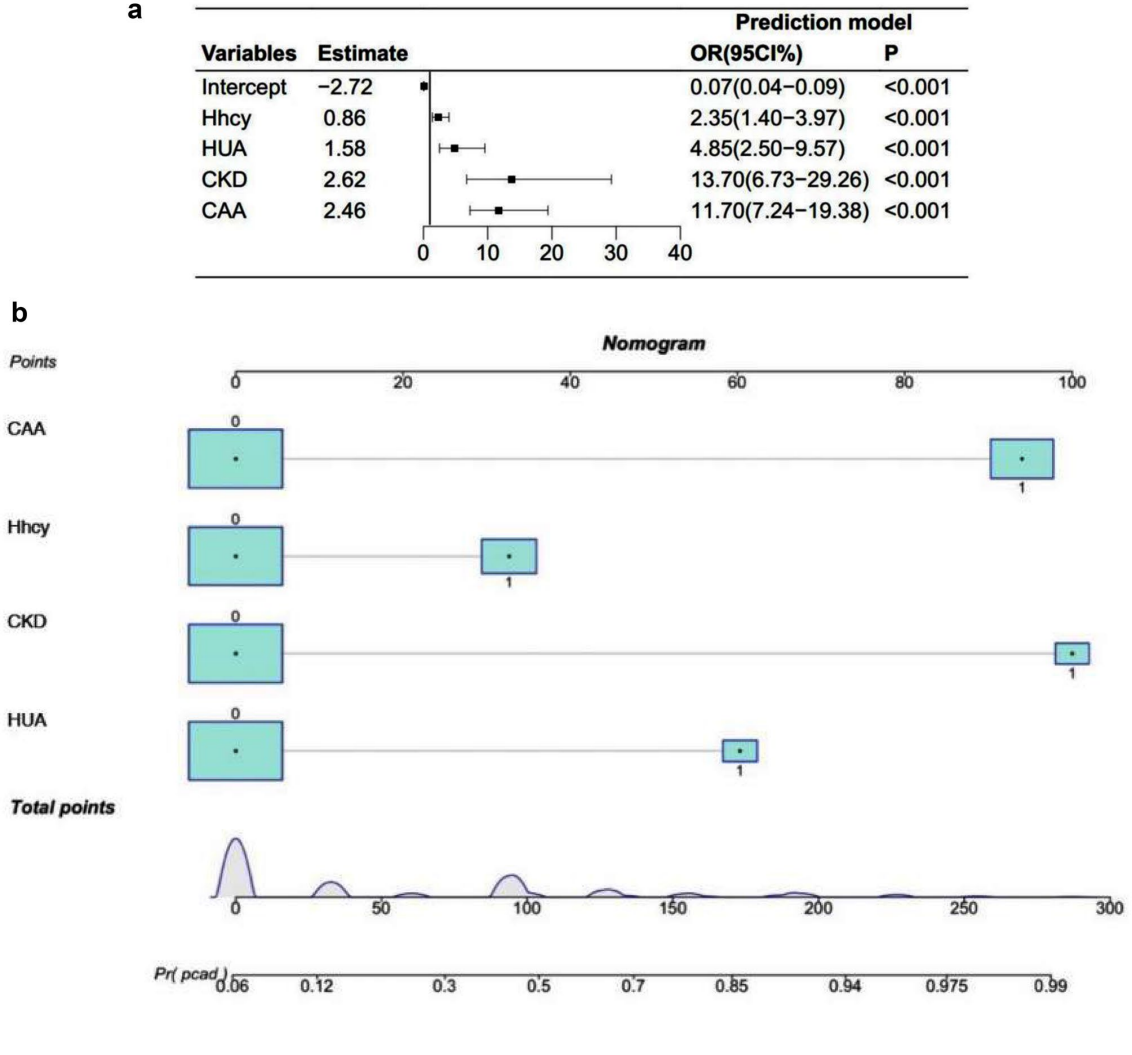
Based on the predictors selected by LASSO. (**a**) A forest plot based on the predictors selected by LASSO. (**b**) PCAD nomogram prediction model

We included PCAD as the dependent variable in the LASSO regression and included 24 variables associated with PCAD in the LASSO regression as independent variables based on a literature review. Dashed vertical lines were plotted for the best values using one standard error, and the best values were selected using 10-fold cross-validation. Four significant indicators with a non-zero coefficient were selected: HUA, CKD, Hhcy and CAA (Supplementary Fig. 1). A PCAD risk prediction model was constructed based on these four predictors (Fig. 2). Then the logistic regression analysis results revealed that CKD(Waldχ^2^=49.10,odds ratio [OR] = 13.70, 95% confidence interval [CI]: 6.73–29.26, *p < 0.001*),HUA(Waldχ^2^=21.35,OR = 4.85, CI: 2.50–9.57, *p < 0.001*),Hhcy(Waldχ^2^=10.46,OR = 2.35, 95% CI: 1.40–3.97, *p < 0.001*) and CAA (Waldχ^2^=96.18,OR = 11.70, 95% CI: 7.24–19.38, *p < 0.001*) were the most important factors affecting the development of PCAD in the patients *(all, p < 0.05*) in Table 2. A model was constructed using logistic regression:

$${{\bf{y}}_{{\bf{model}}}} =- 2.72 + 0.86\cdot{\text{Hhcy}} + 1.58\cdot{\text{HUA}} + 2.62\cdot{\text{CKD}} + 2.46\cdot{\text{CAA}}$$

**Table 2:** Logistic regression analysis of risk predictors of morbidity in patients with PCAD

Intercept and variables	Estimate	SE	Waldχ^2^	Prediction model		Confidence interval(2.5%)	Confidence interval(97.5%)
				*P*-value	Odds ratio		
Intercept	-2.72	0.22	157.44	< 0.001	0.07	0.04	0.09
Hhcy	0.86	0.27	10.46	< 0.001	2.35	1.40	3.97
HUA	1.58	0.34	21.35	< 0.001	4.85	2.50	9.57
CKD	2.62	0.37	49.10	< 0.001	13.70	6.73	29.26
CAA	2.46	0.25	96.18	< 0.001	11.70	7.24	19.38

The variables entering the logistic regression model are used to construct the PCAD nomogram prediction model (Fig. 2).

**Fig. 3:**
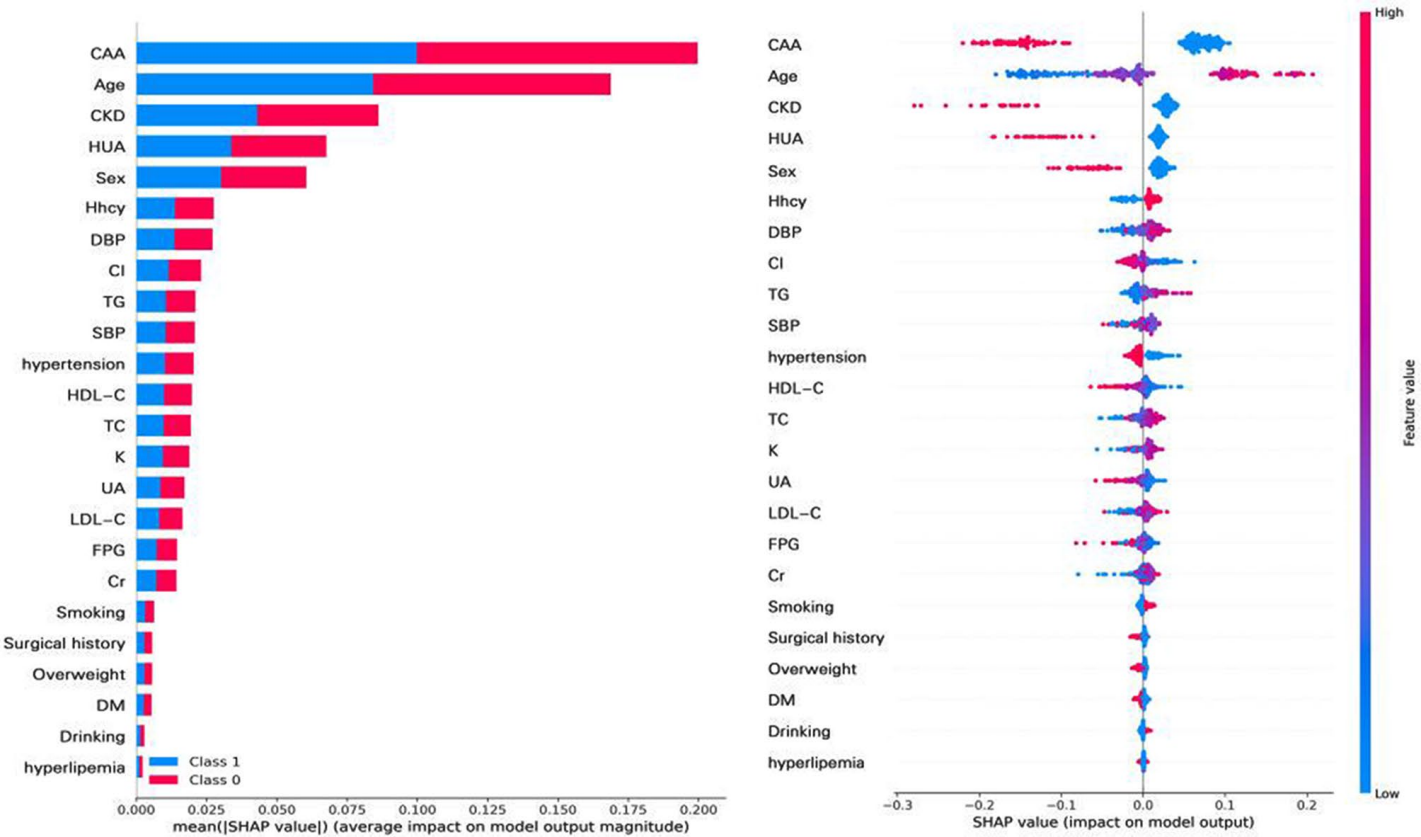
SHAP-based feature importance of RF model

In our RF model, the mtry value represents the number of candidate variables in each node. An appropriate mtry value can improve the classification ability of the model. In RF model, the lowest model error rate was 0.10 when mtry was 10. The ntree parameter refers to the number of decision trees used during modeling. When the number of decision trees is large enough, the error of the model is very stable. In our model, when ntree was 1500, the error rate tends to be stable. The RF model can calculate the degree of influence of each independent variable on the dependent variable and calculate the importance scores according to two different standards. Based on the SHAP method, in order, CAA, age, CKD, HUA and sex had the highest contribution in PCAD prediction (Fig. 3).

**Fig. 4:**
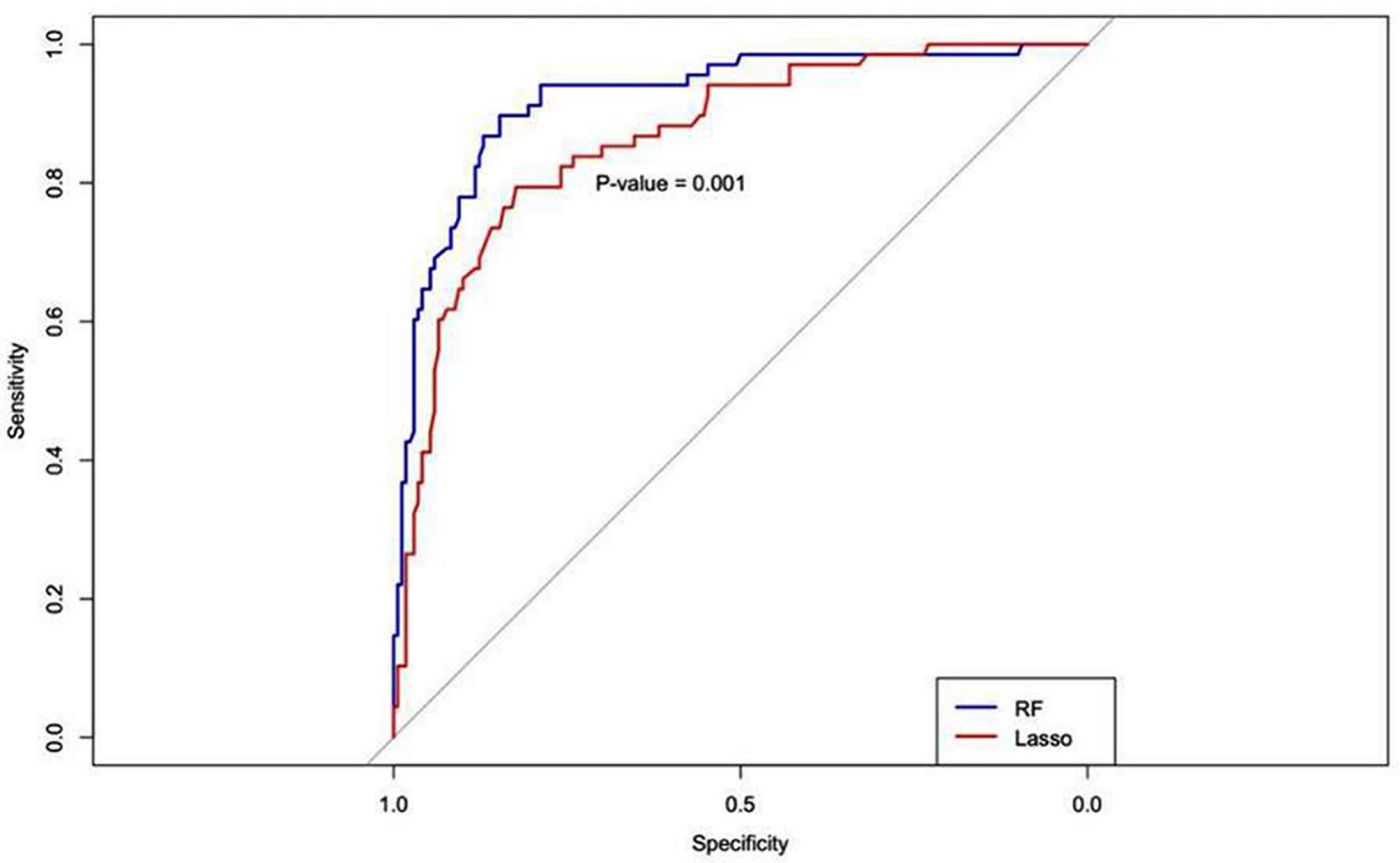
RF versus LASSO Logistic regression models are shown in the ROC curves of the validation set

**Fig. 5:**
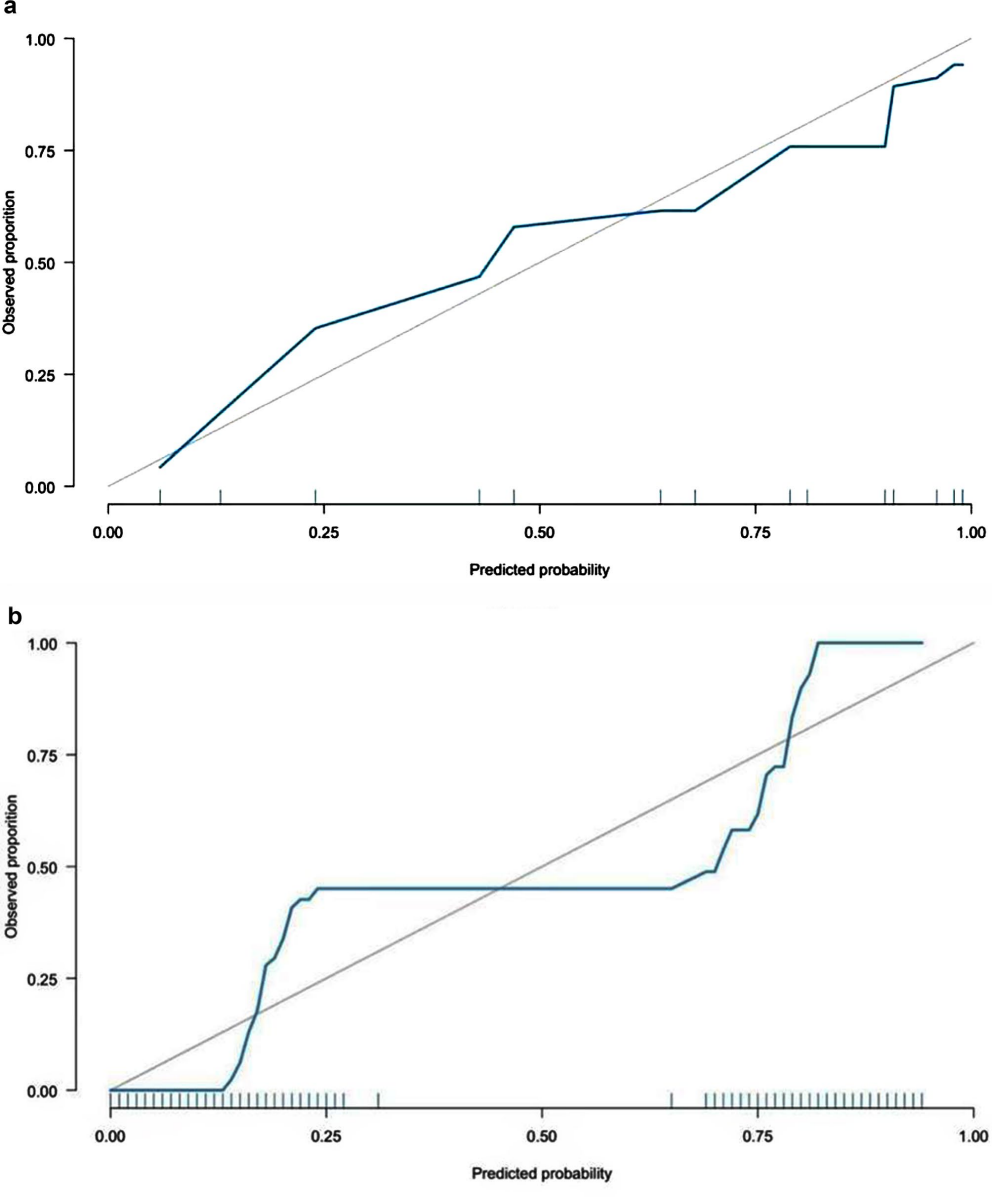
LASSO Logistic regression versus the Calibration curve in the validation set. (**a**) The A LASSO Logistic regression model. (**b**) RF model

HUA, CAA, CKA are significant predictors of PCAD, using the three could differentiate two groups (Supplementary Fig. 2). The specificity, sensitivity, PPV, NPV of the RF model were higher than the LASSO Logistic in the validation set (Table 3). The AUC (Area Under ROC Curve) of the RF model and LASSO Logistic model were 0.91 (95% CI: 0.79–0.88) and 0.84 (95% CI: 0.74–0.84) in the validation set separately. A results of the AUC between the two models showed statistical difference (*Z* = 3.99, *P* < 0.05) (Fig. 4). The two models were internally verified by the bootstrap self-sampling method, and the calibration curve was obtained 50 times (Fig. 5). The calibration curve shows the entirely consistent comparison of each model and the predicted and actual probabilities of the model. The calibration curves of the two models are close to the diagonal line (ideal prediction situation, with a slope of 1), which shows that the prediction ability of the models is acceptable.

**Table 3:** Comparison of the predictive performance of the two models

	Cutoff vale	specificity	sensitivity	Kappa	PPV	NPV
RF	0.39	0.63	0.92	0.55	0.63	0.86
LASSO-Logistic	0.39	0.44	0.93	0.37	0.73	0.81

PPV = positive predictive value;NPV = negative predictive value

## Discussion

The prevalence of PCAD in all CAD was about 28.3% in this study. Both the LASSO Logistic and RF models showed that HUA, CAA and CKD were important predictors of PCAD. In the two models constructed in this study, the discrimination and calibration ability of RF was higher than that of LASSO Logistic models. The accuracy of RF and LASSO Logistic models were 84.0% and 79.4% respectively. Therefore, the RF model has higher application value in PCAD risk prediction.

HUA as a non-traditional high-risk factor of CAD has been confirmed to be involved in the occurrence and development of CAD [27]. Our results showed that HUA is an important predictor in the LASSO and RF models, which is consistent with the results reported by Wang et al [28]. HUA contributes to the development of hypertension, the increase of inflammatory markers, and the impairment of glucose metabolism, which all may promote the occurrence of atherosclerosis or plaque rupture alone or in combination [29]. However, Battaggia et al. reported that HUA is not an independent influencing factor of CAD [30]. Many studies in China have shown that the UA level of patients aged ≥ 40 is positively correlated with CAD, which is similar to the results of our study. However, the relationship between UA level and CAD in patients aged ≤ 35 years is controversial, and it may be impacted by gender. A study of American teenagers showed that increased UA level was related to increased risk of various cardiovascular risk factors, especially in women [31]. Wang et al. reported that the predictive effect of UA on CAD in women is stronger than that in men, both of which are similar to the results of our study [32]. However, other studies report opposing results [33, 34]. In our study, the age of male patients (51.52 ± 0.17) was generally lower than that of female patients (60.45 ± 0.31), thus most of the female patients were postmenopausal. The UA level of postmenopausal women increases significantly, which may be related to the decline of estrogen level after menopause and the loss of estrogen to promote UA excretion, which may lead to endothelial dysfunction [13, 35]. Elevated UA levels and inflammatory markers are associated with many cardiovascular risk factors, including hypertension, hyperlipidemia, and obesity. However, the underlying mechanism of how gender affects HUA is not clear, and the relationship between gender and HUA in adolescent patients requires further exploration.

In the two model of this study, CAA and CKD are important factors of PCAD. The Kidney Disease guideline points out that all the CKD patients have a higher risk of cardiovascular disease, and about 60.0% of CKD patients are accompanied by cardiovascular disease [36, 37]. As the heart and kidney share the same pathophysiological basis.When one organ is damaged, the other organ will also be affected [38]. The mechanism may be that CKD will lead to a significant increase in the content of asymmetric dimethylarginine, an inhibitor of nitric oxide synthase, and accelerate the formation of CAD [39–41]. CKD causes a systemic, chronic proinflammatory state contributing to vascular and myocardial remodeling processes resulting in atherosclerotic lesions, vascular calcification, and vascular senescence as well as myocardial fibrosis and calcification of cardiac valves. Hence CKD lead to an accelerated aging of the cardiovascular system. A large sample study on the prevalence of CKD among young patients with cardiovascular disease showed, mortality risk of the patients aged 18–50 years has been increased to 3.6 times compared to last year due to the CKD [42]. Therefore, abnormal renal function and CAA pay a positive role in predicting the occurrence of PCAD.

The evaluation and comparison results of the two models show that the performance of the RF model in this study is better than that of the LASSO Logistic model. A Chinese study used RF and LASSO Logistic regression to predict the hospitalization expenses of patients with chronic renal failure, and the results showed that the prediction performance of RF model was better than that of LASSO Logistic model [43]. Another study constructed the prognosis model of diffuse large B-cell lymphoma, which indicated that the prediction performance of LASSO Logistic model was better than that of RF model [44]. Therefore, there is no accurate conclusion about the prediction performance of the two algorithms. The possible reasons of different research conclusions are the RF does not limit the correlation between variables, while the exclusion of highly correlated variables in LASSO Logistic modeling often excludes important variables highly correlated with response endpoints. Therefore, the LASSO model may reduce the prediction performance. Some researches show that it is more important to screen variables correctly than modeling learning algorithm [45].

There are several limitations in this study. First, the two models have not been externally validated. In the future, we can collect data from different hospitals to obtain transportability the model by externally validate. Second, the data of this study collected clinical cases,so other biochemical indicators could not be analyzed. Finally, only two algorithms are used to build the model. In the future, other machine algorithms can be used to obtain more accurate models.

## Conclusion

HUA, CAA, CKA are significant predictors of PCAD in this study. The two predictive models are established for predicting the occurrence of PCAD. RF model has a higher accuracy. Using the PCAD risk prediction model, early screening of high-risk groups for PCAD can be effectively conducted, and personalized intervention plans for patients can be developed to prevent and delay the occurrence of PCAD. Such screening would provide primary prevention for PCAD patients and would improve the allocation of national medical and health resources.

### Electronic supplementary material

Below is the link to the electronic supplementary material.

Supplementary Material 1

## Data Availability

The datasets used and analyzed during the current study are available from the corresponding author on reasonable request.The use of data in this study is limited, and the data set can be obtained from the corresponding author (Yikang Xu) according to reasonable requirements. Researchers or readers can send emails to corresponding author’s mailbox, and we will share the data without stint.
