# Supplementary material for: Comparison of LASSO and random forest models for predicting the risk of premature coronary artery disease
Source: BMC Med Inform Decis Mak. 2023 Dec 20;23:297. doi: 10.1186/s12911-023-02407-w (PMC10734117; doi:10.1186/s12911-023-02407-w)
Supplement: Supplementary file 1 — Supplementary Material 1 [file 12911_2023_2407_MOESM1_ESM.docx]

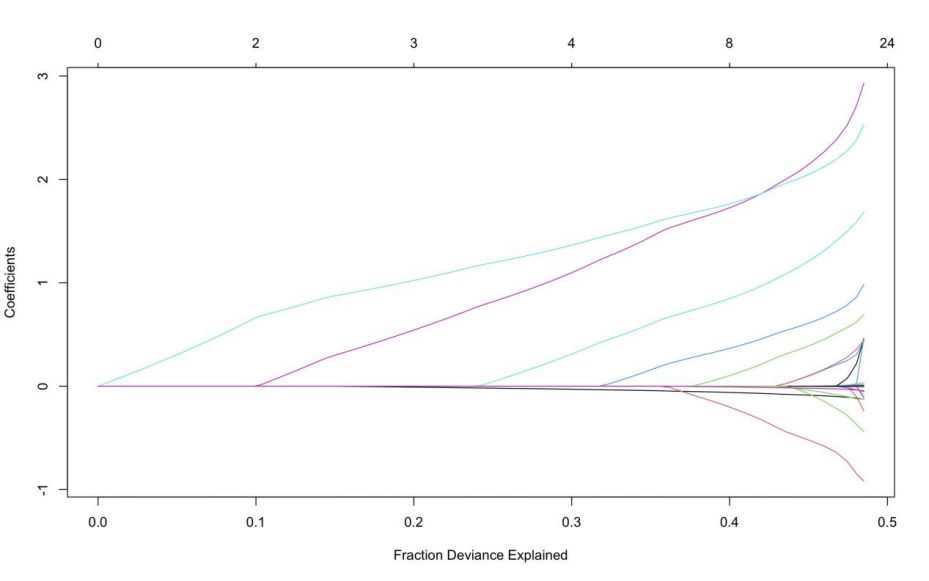


a.


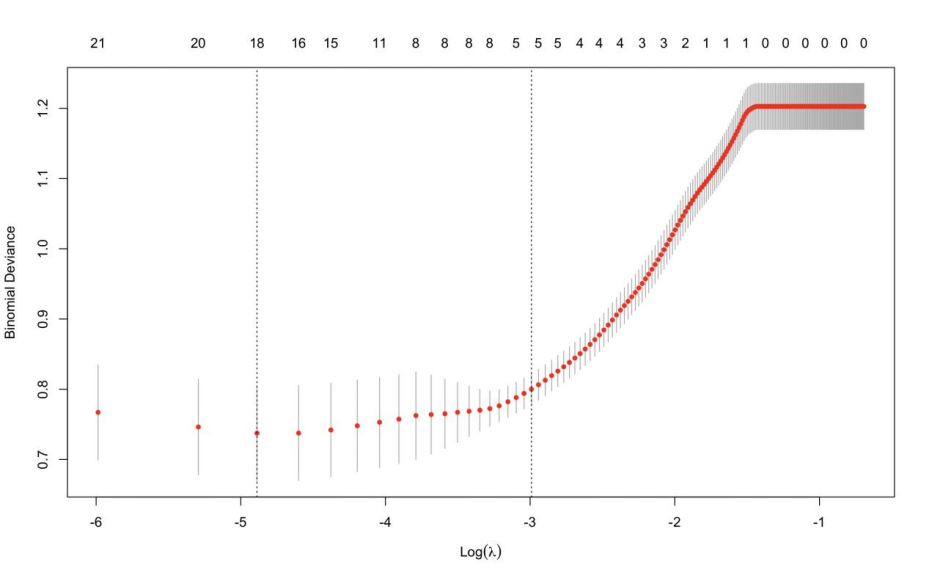


b.

Supplementary Figure 1：Screening of characteristic variables based on LASSO regression. **a** the change characteristics of the variable coefficients. **b** the best process of screening by a 10-fold cross-validation method in the LASSO model, with five variables selected by log (λ) = -1.30, which is the most streamlined model.
